# Assessing invertebrate herbivory in human‐modified tropical forest canopies

**DOI:** 10.1002/ece3.7295

**Published:** 2021-03-26

**Authors:** Julia Rodrigues Barreto, Erika Berenguer, Joice Ferreira, Carlos A. Joly, Yadvinder Malhi, Marina Maria Moraes de Seixas, Jos Barlow

**Affiliations:** ^1^ Setor de Ecologia e Conservação Universidade Federal de Lavras Lavras Brazil; ^2^ Programa de Pós‐Graduação em Ecologia do Instituto de Biociências da USP Universidade de São Paulo São Paulo Brazil; ^3^ School of Geography and the Environment Environmental Change Institute University of Oxford Oxford UK; ^4^ Lancaster Environment Centre Lancaster University Lancaster UK; ^5^ Embrapa Amazônia Oriental Belém Brazil; ^6^ Departamento de Biologia Vegetal Instituto de Biologia Universidade Estadual de Campinas Campinas Brazil

**Keywords:** Amazon rainforest, biodiversity and ecosystem functioning, environmental gradient, folivory, forest degradation, herbivore interactions, herbivory, plant, tropical forest

## Abstract

1. Studies on the effects of human‐driven forest disturbance usually focus on either biodiversity or carbon dynamics but much less is known about ecosystem processes that span different trophic levels. Herbivory is a fundamental ecological process for ecosystem functioning, but it remains poorly quantified in human‐modified tropical rainforests.
2. Here, we present the results of the largest study to date on the impacts of human disturbances on herbivory. We quantified the incidence (percentage of leaves affected) and severity (the percentage of leaf area lost) of canopy insect herbivory caused by chewers, miners, and gall makers in leaves from 1,076 trees distributed across 20 undisturbed and human‐modified forest plots in the Amazon.
3. We found that chewers dominated herbivory incidence, yet were not a good predictor of the other forms of herbivory at either the stem or plot level. Chewing severity was higher in both logged and logged‐and‐burned primary forests when compared to undisturbed forests. We found no difference in herbivory severity between undisturbed primary forests and secondary forests. Despite evidence at the stem level, neither plot‐level incidence nor severity of the three forms of herbivory responded to disturbance.
4. *Synthesis*. Our large‐scale study of canopy herbivory confirms that chewers dominate the herbivory signal in tropical forests, but that their influence on leaf area lost cannot predict the incidence or severity of other forms. We found only limited evidence suggesting that human disturbance affects the severity of leaf herbivory, with higher values in logged and logged‐and‐burned forests than undisturbed and secondary forests. Additionally, we found no effect of human disturbance on the incidence of leaf herbivory.

## Graphical abstract

Our large‐scale study of canopy herbivory in Amazonian forests found only limited evidence suggesting that human disturbance affects leaf herbivory.

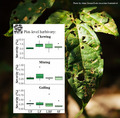

## 1 INTRODUCTION

Tropical rainforests are important reservoirs of biodiversity (Bradshaw et al., 2009) and provide a myriad of ecosystem services that are vital to humanity, including carbon sequestration and storage (Berenguer et al., 2014), as well as rainfall generation (Spracklen et al., 2012). Amazonia, the largest tropical rainforest in the world, holds 16,000 tree species (Ter Steege et al., 2020) and stores approximately 86 Pg carbon (Saatchi et al., 2007). Despite their great importance, Amazonian forests are under threat from human activities such as selective logging and wildfires (Aragão et al., 2018; Barlow et al., 2016; Brancalion et al., 2018; Bullock et al., 2020). While much effort has gone into understanding how human disturbances affect either the biodiversity or the carbon dynamics in these disturbed forests (Barlow et al., 2016; Berenguer et al., 2015; Lennox et al., 2018; Robinson et al., 2015), much less is known about the impacts on ecosystem processes.

Herbivory is a fundamental ecosystem process across the world, involving over half of all terrestrial species (Zangerl et al., 2002). It acts as an important pathway for energy flow from plants to upper trophic levels (Agrawal, 2000; Coley et al., 1996; Hempson et al., 2015) and has a strong influence on both the quantity and the quality of organic material transferred to the soil, thus affecting nutrient cycling (Bardgett & Wardle, 2003; Metcalfe et al., 2014). Yet our understanding of herbivory rates in tropical forest systems remains limited, especially in rainforests (Kristensen et al., 2020). For example, although it was believed that tropical ecosystems experience higher rates of herbivory than temperate ones (Coley et al., 1996), such patterns have not been confirmed by more recent global assessments (Kozlov et al., 2015).

There are at least three key knowledge gaps that limit our understanding of herbivory patterns in tropical rainforests. First, no studies have examined how herbivory rates respond to human disturbance, even though these could be crucial for refining important estimates of both carbon and nutrient cycling across much of the remaining tropical forest biome (Metcalfe et al., 2014). There are strong a priori reasons to think that herbivory may change due to human influence, given disturbance can alter insect densities (Knight & Holt, 2005), resource quantity (McNaughton et al., 1989), resource quality (Coley et al., 1985), plant defenses (Coley, 1987; McIntyre et al., 1999), and top‐down control of herbivores (Dodonov et al., 2016). Second, many studies focus on leaf loss from chewing invertebrates (e.g., Fagan et al., 2005; Silva et al., 2012; Wolf et al., 2008), but do not consider other forms of invertebrate‐mediated herbivory that are widespread in tropical forests, such as miners and gall makers. This could be an important omission, as both of these groups have severe impacts on trees, shrubs, and crops elsewhere (Cocco et al., 2015; Kozlov et al., 2017; Valladares et al., 2006). Finally, most of our knowledge of herbivory in tropical rainforests come from sampling in the understorey (e.g., Aide, 1993; Aldea et al., 2006; Angulo‐Sandoval et al., 2004), with few studies measuring herbivory in tropical canopy leaves (Bixenmann et al., 2016; Darrigo et al., 2018), most likely due to the difficulty of accessing such heights. This is important as the vast majority of leaf area is in the canopy, and processes measured in dark understories characterized by slow growing plants are unlikely to reflect those that occur in tree canopies.

To address these knowledge gaps, we assessed herbivory incidence and severity from chewers, miners, and gall makers in canopy leaf blades from 1,076 individual trees covering 250 species. Trees were located in 20 forest plots in the Tapajós region of eastern Amazonia, distributed in undisturbed primary forests, logged primary forests, logged‐and‐burned primary forests and secondary forests. To address the methodological knowledge gap that most studies only assess chewing when measuring herbivory, we ask: (a) How prevalent is chewing relative to mining and gall‐forming herbivory?, (b) can one form of herbivory (e.g., chewing) be used as a reliable proxy of other forms of herbivory at either the stem or plot level, and are these relationships consistent within forest disturbance classes? We then examine how herbivory rates respond to human disturbance, asking (c) does stem‐level or plot‐level herbivory vary between forest disturbance classes? We address these questions using both stem and plot level assessments of herbivory, matching the scale of assessments used in previous studies (e.g., Schowalter, 2016). We compare our estimates of herbivory with those published in the literature, and discuss which factors contribute to the variation in rates of herbivory.

## 2 MATERIALS AND METHODS

### 2.1 Study area and forest disturbance classes

This study was conducted during the dry season of 2015 in a region of eastern Amazonia that encompasses the municipalities of Santarém, Belterra, and Mojuí dos Campos (hereafter Santarém), in the state of Pará, Brazil (Figure S1). Data were sampled across 20 plots (10 × 250 m, 0.25 ha) distributed along the following four forest classes: undisturbed primary forests (*n* = 5), logged primary forests (*n* = 5), logged‐and‐burned primary forests (*n* = 5), and secondary forests recovering after agricultural abandonment (*n* = 5). These 20 plots (Table S1) were selected from a larger set of 108 forest plots established in 2010, which were located in evergreen nonflooded forests. Plots were placed at least 100 m from forest edges, to avoid edge effects, and >1.5 km apart, to avoid spatial autocorrelation (see Gardner et al., (2013) for further explanation of sampling design). The subset of 20 plots were chosen as they were (a) logistically possible to be sampled (given permission to work on private lands was required), (b) balanced in terms of sample sizes for the four forest classes, (c) spatially distributed to avoid autocorrelation as much as possible, and (d) to pair more than one disturbance type within the same catchments as much as possible. The average wood densities of plots in each disturbance class were also broadly representative of values derived from a much larger sample size (Berenguer et al., 2018), suggesting they reflect average conditions for the region. Forest classes were defined using a combination of field assessments of evidence of previous human disturbance (e.g., logging debris, charred stems) and an analysis of canopy disturbance, deforestation, and regrowth in a 20‐year chronosequence of satellite images (for more information about forest classification see Gardner et al., 2013).

### 2.2 Sampling and defining stem and plot level herbivory

To assess foliar herbivory, we first selected all tree species ≥10 cm diameter at breast height that contributed to 80% of the basal area of each plot. The species selection criterion was based on Grime's “mass‐ratio hypothesis”, which proposes that the rate of an ecosystem function is primarily determined by the characteristics of the dominant plant species (Grime, 1998). In each plot, we sampled up to three individuals from the selected species—often species were only represented by doubletons or singletons. In the few cases in which a species had more than three individuals present in a plot, we sampled the three largest ones. For each individual stem, a tree climber using a 10‐m pruner selected a branch composed of mature leaves that were fully exposed to sunlight. In each branch, we assessed incidence of the three forms of herbivory and severity of mining and galling in all leaves (Figure S2). We defined herbivory incidence at the stem level as the number of leaves affected by each form of herbivory (i.e., chewers, miners, and gall makers) divided by the total number of leaves present in a given branch. This was analyzed as a proportion, but is expressed as a percentage in the text and figures. For all analyses at the plot level, we used a community weighted mean, with herbivory weighted by each species relative contribution to the plot's basal area.

Herbivory severity was defined as the percentage of leaf area lost to each herbivory type. To ensure comparability to previous studies (e.g., Schowalter, 2016), we first calculated stem‐level severity. We did this by averaging the percentage of lamina loss across all leaves (including damaged and undamaged ones) in an individual. To measure herbivory severity caused by miners and gall makers, each leaf was visually assessed and assigned to one of the following damage classes: intact leaves, 0.01–1, 1–5, 5–25, 25–50, 50–75, and 75%–100% (following Alliende, 1989). In total, we evaluated the herbivory severity of miners and gall makers across 196,388 leaves.

For chewers, a single observer randomly selected 30 chewed leaves per individual (or the total number of damaged leaves if the total was ≤30). In total, 29,586 leaves were selected and taken to the laboratory for image analysis. To measure the percentage of area lost, we first used a graphics software (Photoshop CS, Adobe Systems Incorporated) to manually draw the outline of all leaves with damaged edges, so we could recreate the original leaf area (i.e., prior to damage). Then, using imagery software (ImageJ; NIH, version 1.49u), we calculated leaf area considering herbivory (i.e., including all holes; Ah, cm^2^) and then adjusted the image to fill the damaged area in order to estimate the original leaf area (i.e., prior herbivory; Anh, cm^2^). The difference between leaf area prior (Anh) and post (Ah) herbivory was divided by the original leaf area (Anh) to calculate the percentage loss of leaf area (H), as described in Metcalfe et al. (2014). Chewing severity at the stem level was calculated in the following steps. First, if the total number of chewed leaves exceeded 30 (which was only in 5% of cases) we applied the mean percentage of lamina lost from our scanned sample of 30 leaves to all other chewed leaves. Second, we calculated the average percentage of lamina area lost to herbivory across all leaves for that stem. As an example, the average chewing severity for a stem would be 25% if the stem had 100 leaves in total, 50 of those had signs of chewing herbivory, and our sample of 30 leaves revealed an average area loss of 50%. For a full schematic description of the sampling design, see Figure S2. For plot‐level analyses of severity, we used community weighed means.

### 2.3 Statistical analysis

To investigate whether herbivory incidence varied between the three forms—chewing, mining, and galling—we ran a Kruskal–Wallis test, regardless of forest disturbance. Then, to assess which form varied from each, we ran a multiple comparison test (“kruskalmc” function, R package *pgirmess*). To assess whether one form of herbivory could represent other(s), regardless of forest disturbance class, we tested the correlation between all herbivory forms at both the stem and plot level. At the stem level, we used correlation coefficients calculated for each individual observation, while for the plot level, we extracted the mean Spearman's correlation coefficient per plot. Correlation tests were carried out using the “ggpairs” function of the R package *GGally* (Schloerke et al., 2018). To explore whether correlation patterns at both stem and plot levels were held across the different forest classes, we also compared Spearman's correlation values along the forest classes.

To examine how herbivory is influenced by disturbance classes, we used general linear mixed‐effect models (GLMM). Our response variables have different non‐Gaussian distributions: Herbivory incidence had a binomial distribution, while herbivory severity presented a Tweedie (family of exponential distributions in the “*glmmTMB*” package) distribution. We therefore used models with error structures appropriate for each response variable. We used the sampling site, and the stem's species and family as random effects. As suggested by Bolker (2015), to deal with overdispersion in incidence and severity models, we also included stem id as an observation‐level random effect (OLRE). OLRE allows extra variance in the response observations, which is not accounted for in other terms in the model, by using a random effect with a unique level for every data point (Harrison, 2014, 2015). We tested for potential spatial autocorrelation between models running Moran's I test from the R package *DHARMa* to make sure our samples were independent. We found no evidence of spatial autocorrelation (Table S3). After defining the models, to examine whether and how either stem‐ or plot‐level herbivory of the three different forms of herbivory (i.e., chewers, miners, and gall makers) varied across forest classes, we tested the model containing forest classes against its respective null model by running an analysis of variance (ANOVA). After, we used pairwise interactions through the “Test Interactions” function (R package *phia*) to check whether variance of herbivory was significant between each forest disturbance class pairwise. All analyses were carried out in Rstudio (linked to R version 3.6.1 GUI 1.68 Mavericks build).

## 3 RESULTS

### 3.1 Prevalence of the different forms of herbivory

Stem‐level herbivory incidence was extremely variable, with 0%–100% of leaves affected per stem (mean ± *SD *= 42.5% ± 33; *n* = 1,076 stems), while stem‐level herbivory severity ranged from 0% to 53.9% (mean ± *SD* = 2.5% ± 4; *n* = 1,076 stems). All stems sampled presented at least one form of herbivory damage, and only 29.9% of all leaves presented no damage at all.

**FIGURE 1:**
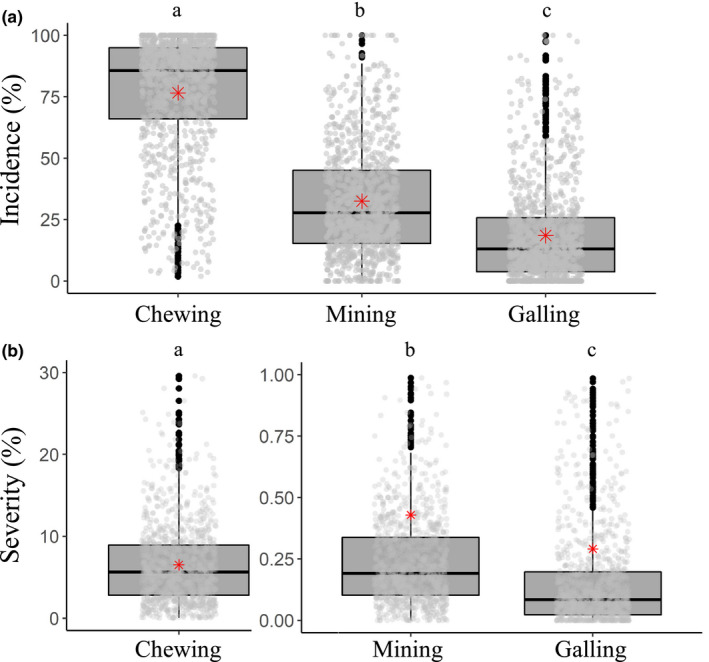
Comparison between herbivory incidence (a) and severity (b) caused by chewers, miners, and gall formers in 1,076 stems. Different letters on top of boxes represent significant differences at *p* < .05 following a Kruskal–Wallis test. Boxplots display the distribution of the data, showing the first and third quartiles, whiskers extend up to 1.5 times the interquartile range. Light gray dots represent actual data distribution, black dots are outliers, and red asterisks represent mean values. Notice that the *y*‐axes in panel b are on different scales

Chewing was the most prevalent form of herbivory, with 76.6% ± 24 of stems affected by it, followed by mining (mean ± *SD* = 32.5% ± 22) and galling (mean ± *SD* = 18.6% ± 19; Figure 1). These differences between stem‐level herbivory incidence were highly significant (Kruskal–Wallis *χ* ^2^ = 1,636.4, *df* = 2, *p* ≤ .001). The severity of chewing was also more prevalent than other forms of herbivory (mean ± *SD* = 6.7% ± 5.4), followed by mining (mean ± *SD *= 0.42% ± 1.4) and galling (mean ± *SD* = 0.29% ± 1.2; Figure 1b). These differences were highly significant (*χ* ^2^ = 4,295.4, *df* = 2, *p* ≤ .001).

### 3.2 Can one form be used as a reliable proxy of other forms of herbivory at either the stem or plot level?

**FIGURE 2:**
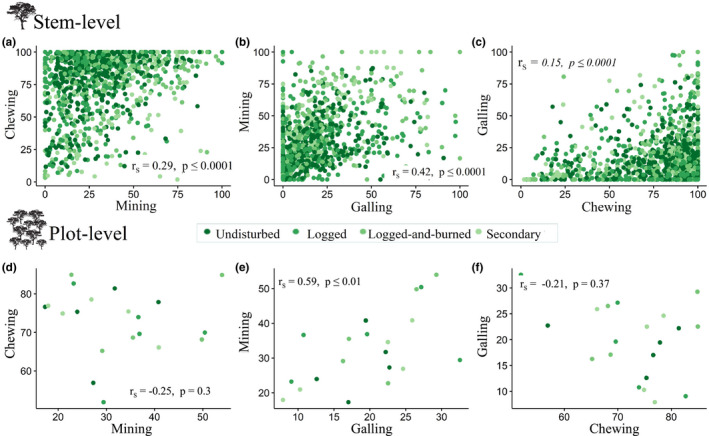
Correlation between the different forms of herbivory incidence at the stem (a–c) and plot level (d–f). Notice that *y*‐axes are not on the same scale

For all stems together, there were significant correlations between the incidence of all three forms of herbivory (*p* ≤ .0001 in all cases, *n* = 1,076; Figure 2a–c), although coefficients were all below 0.5 (*r*_S_ range from .15 to .42, *n* = 1,076). Correlations between stem‐level severity were also significant (*p* ≤ .01 in all cases, *n* = 1,076), although the correlation coefficients were lower than for incidence (*r*_S_ range from .08 to .22, *n* = 1,076; Figure S4a‐c). We also explored the relationships between herbivory forms within each of the forest disturbance classes at the stem level (Figure S5). In this analysis, only the incidence of mining and galling were highly correlated within secondary (*r*_S_ = .6, *p* ≤ .001, *n* = 146) and logged‐and‐burned forests (*r*_S_ = .5, *p* ≤ .001, *n* = 229; Figure S45f).

For incidence at the plot level, only mining and galling were significantly correlated (*r*_S_ = .59, *p* ≤ .01, *n* = 20; Figure 2e). For severity, none of the herbivory forms were significantly correlated at the plot level (*r*_S_ ranged from −.14 to .31, *n* = 20; Figure S4d‐f). Plot‐level herbivory within each of the forest disturbance classes presented very high correlation between the incidence of mining and galling in undisturbed (*r*_S_ = .9, *p* ≤ 1, *n* = 5) and secondary forests (*r*_S_ = .7, *n* = 5; Figure S45m), but these correlations were nonsignificant (*p* ≥ .5). Chewers’ incidence was negatively correlated with galling within logged‐and‐burned forests (*r*_S_ = −.9, *n* = 5; Figure S5l), but not in other forest disturbance classes. For severity, the percentage of leaf area affected by chewers was highly negatively correlated with leaf area affected by gall makers in undisturbed plots (*r*_S_ = −.1, *p* ≤ .5, *n* = 5) and positively correlated in secondary forests (*r*_S_ = .7, *n* = 20; Figure S5h).

### 3.3 Influence of human disturbance on herbivory levels

**FIGURE 3:**
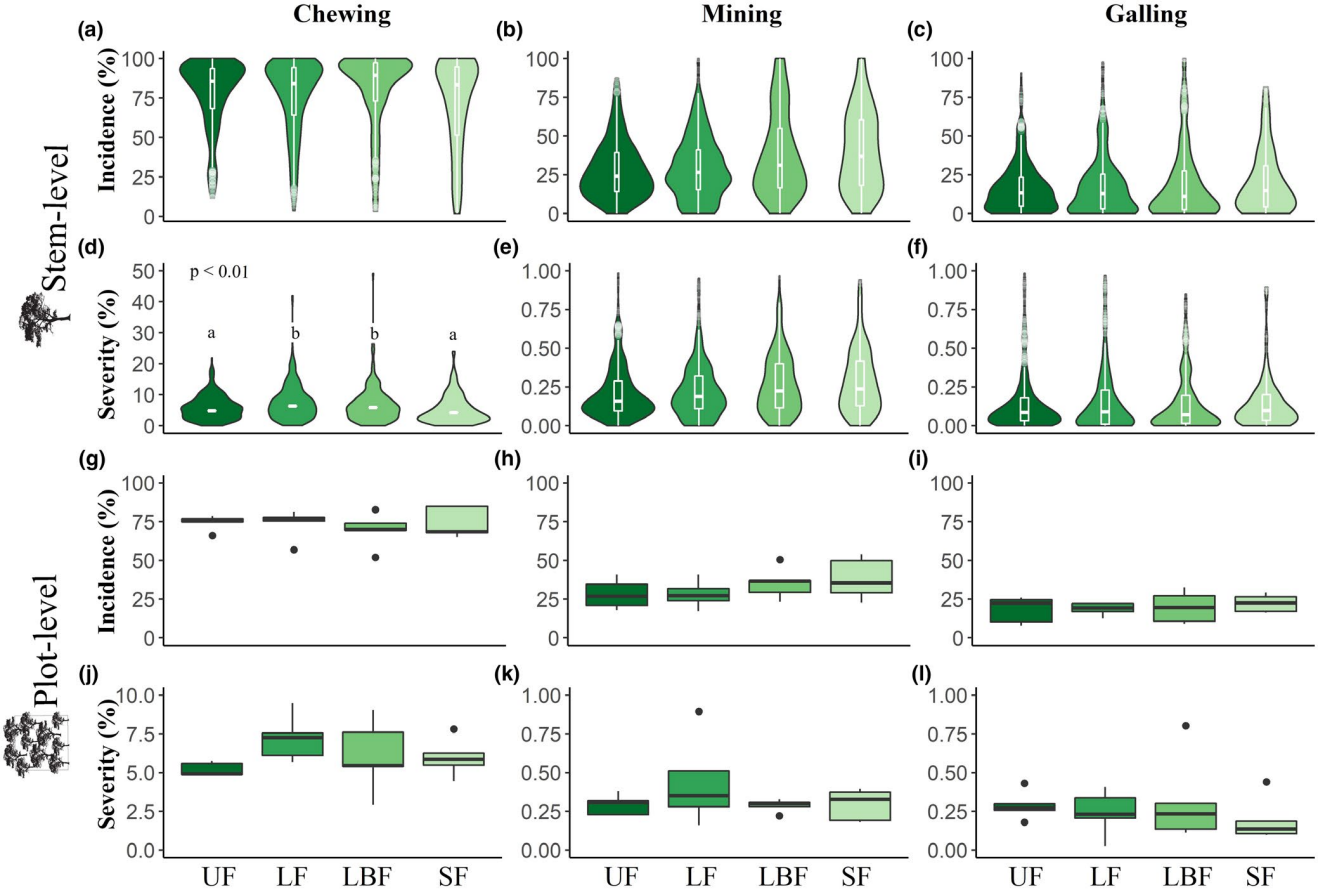
Herbivory levels across a gradient of forest disturbance. Stem‐level incidence (a–c) and severity (d–f); plot‐level incidence (g–i) and severity (j–l). Invertebrate‐mediated herbivory forms are represented per column: chewing (a, d, and g), mining (b, e, and h), and galling (c, d, and i). Notice that severity plots’ *y*‐axes (d–f, j, and k) are not on the same scale. Both the box and violin plots display the distribution of the raw data, except for the only signifficative result (stem‐level chewing severity, (d) where we plot model predictions instead. Boxplots (including white ones within violin plots area) represent first and third quartiles, whiskers extend up to 1.5 times the interquartile range, data points beyond that threshold are plotted individually. Plots were colored according to forest classes, scaling from lighter to darker green representing the disturbance gradient: UF, undisturbed forests; L, logged forests; LBF, logged‐and‐burned forests; SF, secondary forests

At the stem level, there were no significant differences in the incidence of herbivory across different forest disturbance classes (Figure 3a–c). For stem‐level severity, only chewing was significantly different across disturbance classes (*p* < .01**, *χ* ^2^ = 15.429, *df* = 3; Figure 3d), with higher values in logged (mean ± *SD *= 7.8% ± 1) and logged‐and‐burned (mean ± *SD* = 7.1% ± 1) forests than in undisturbed and secondary forests (mean ± *SD* = 5.9% ± 1 and 5.3% ± 1, respectively). At the plot level, none of the community weighted mean assessments of herbivory incidence or severity—that is, for chewers, miners, and gall makers—were different across forest classes (Figure 3g–l).

## 4 DISCUSSION

We present the results of a large‐scale herbivory study, assessing a total of 196,388 leaf blades across 1,076 individual trees distributed in 20 human‐modified forest plots. Chewing incidence was very high across trees (Figure 1) and was found to be the most dominant form of invertebrate‐mediated herbivory, reflecting trends observed across the Neotropics (Novotny et al., 2010; Vasconcelos, 1999). Yet, although chewing is a common measure of total leaf herbivory in many studies (e.g., Schowalter, 2016), it was not a good predictor of other forms of herbivory. We discuss our findings by examining the influence of human‐induced disturbance on herbivory, the often‐overseen impact of leaf miners and gall makers on plants, and the variation in rates of herbivory between different studies, including those that focused on understorey sampling.

### 4.1 Does human disturbance increase leaf herbivory?

We expected herbivory levels to be higher in disturbed forests as these are dominated by pioneer plant species (Laurance et al., 2006), which tend to be more palatable to invertebrates due to lower leaf thickness and less amounts of phenolic compounds (Coley, 1983). Our results provided low support for this expectation—neither stem‐level herbivory incidence or plot‐level incidence and severity presented any significant differences between forest classes for any of the herbivory forms. The only significant variation we found was for stem‐level severity of chewers, which was marginally higher in the two disturbed primary forest classes (Figure 3d).

The lack of strong differences in herbivory may in part be explained by the high level of variation found within the logged and logged‐and‐burned categories, which likely encompassed different recovery times since the disturbance event, different disturbance intensities and different disturbance extents. However, the low level of chewing severity in secondary forests requires a different explanation, as these regenerating forests were all of a similar age (≥18 years old). Furthermore, they were dominated by pioneer species (e.g., *Vismia*, *Annona,* and *Bellucia* genera) that are known to be palatable (Guimarães et al., 2014; Leal et al., 2014; Tabarelli et al., 2012). It is likely that these pioneer plant species compensate for higher damage by having a faster turnover rate of leaves, which would reduce the rate of herbivory detected at the leaf level (Agrawal, 2000).

It may be that top‐down control, in the form of predation pressure on herbivores from vertebrate or parasitoids, could help explain the lack of strong differences in herbivory across disturbance classes. Although insectivorous birds are generally less abundant in human‐modified forests (Moura et al., 2016), leaf gleaning insectivorous birds actually increase in richness in highly disturbed forests (Barlow & Peres, 2004) and could be controlling leaf herbivores. Parasitoids also have strong effects on herbivores (Hawkins et al., 1997), and these are mostly generalists (Lewis et al., 2002) and are well adapted to human‐modified environments, including forests of differing management intensities (Gossner et al., 2014) and landscape heterogeneity (Molina et al., 2019).

### 4.2 Beyond chewers: the importance of other forms of herbivory

One of most commonly used approaches employed to assess herbivory across tree communities involves quantifying leaf area loss (e.g., Metcalfe et al., 2014; Sobek et al., 2009; Souza et al., 2013; Vasconcelos, 1999; Visakorpi et al., 2018). We show that chewers dominate the herbivory signal; for example, the community weighted plot‐level severity of chewing was 9.9 times higher than mining and 23.5 times higher than gall forming, and plot‐level incidence levels were 2.2–3.7 higher than mining and gall‐forming herbivory. Although these headline differences suggest that gall‐forming and internal feeding herbivores can be ignored in assessments of herbivory in tropical forests, there are three reasons why they may also deserve greater attention. First, different forms of herbivory are weakly correlated either when analyzing the full dataset or when looking within each forest class (Figure S4), so chewing herbivory cannot be used as a reliable proxy of mining or gall‐forming herbivores. Second, studies only measuring the leaf area affected may miss key energetic costs for the host plant from gall‐forming or mining herbivores (Giron et al., 2016; Tooker & Giron, 2020). Third, overlooking miners and gall formers can limit our understanding of the relative importance of top‐down and bottom‐up controls (Vidal & Murphy, 2018), as they differ ecologically and are regulated by different top‐down processes (Ohgushi et al., 2012; Zvereva et al., 2020). For example, external feeding habits (e.g., chewers) have increased predation risk due to the exposure and vulnerability at the leaf surface (Kaplan et al., 2014; Schmitz et al., 1997), while miners are less susceptible to predation than external feeders (Hawkins et al., 1997).

Finally, our assessment of different forms of herbivory was not comprehensive. Although they belong to internal feeding guilds, we did not assess sap‐sucking insects (e.g., aphids). However, these are not thought to be common in tropical forests (Dixon et al., 1987) and as such seem unlikely to change the overall results. We also did not estimate vertebrate leaf herbivory, such as that caused by arboreal leaf‐feeding mammals (e.g., sloths, howler and spider monkeys, Chiarello, 1998; Lopez et al.,^2005^; Mittemeier & can Roosmalen, 1981; Urbani & Bosque, 2007) and even birds (Kays & Allison, 2001). The severity of vertebrate herbivory in the canopy remains an important knowledge gap (Metcalfe et al., 2014), especially in human‐modified forests.

### 4.3 Understanding variation in rates of herbivory

Across the Amazon, herbivory levels present a great variation between seedlings and understorey trees; while incidence is much less reported, severity ranges from 1% until 50% of leaf area loss (Benitez‐Malvido et al., 1999; Darrigo et al., 2018; Julião et al., 2017; Massad et al., 2013; Metcalfe et al., 2014; Poorter et al., 2004; Vasconcelos, 1999). Our results are toward the low end of the severity range, with an average 6.7% of leaf area loss by chewers. This large variation in herbivory levels both within and between studies can be due to a number of factors, including plot altitude and topography (Julião et al., 2017; Metcalfe et al., 2014), as well as human‐driven disturbances to the system (Massad et al., 2013). But one of the most likely and important sources of variation is related to the different methods used to assess herbivory incidence and severity. For example, although time consuming and difficult to implement at scale, studies that track herbivory through time using marked leaves are able to measure leaf herbivory rates (Aide, 1993; Lowman, 1984), and usually return estimates three to five times higher than those based on discrete measurements (Lowman, 1984, 1985). Yet single‐census assessments of herbivory levels are the most widely used and time‐ and cost‐effective method of herbivory sampling—and tracking leaves becomes even more complex when assessing forest canopies at scale. Herbivory research could advance if it can identify reliable scaling factors that allow comparisons to be made between different methods.

The forest strata assessed can also explain some of the differences in herbivory rates. Most studies on Neotropical forests examining herbivory damage have focused on seedlings or understorey trees (e.g., Angulo‐Sandoval et al., 2004; Eichhorn et al., 2007), with very few collecting leaves from the canopy (e.g., Fáveri et al., 2008; Ruiz‐Guerra et al., 2010; Weissflog et al., 2018). There is some evidence that herbivory patterns seem to vary between understorey studies and our canopy research. For example, studies in Western Amazonia found that between 0.6% and 10% of all sampled individuals of seedlings and understorey trees presented galls (Julião et al., 2017; Vasconcelos, 1999), while we found galling to be present in 83% of our stems. In the same region, the incidence of miners and chewers was found in 1.5% and 73% of understorey trees, respectively (Vasconcelos, 1999). While we found a higher incidence of miners in canopy leaves (32.5%), the incidence of chewers was very similar to those of understorey trees (76.6%). The percentage of leaves presenting a complete absence of herbivory signs were also similar between understorey and canopy trees—while in understorey trees 24% of leaves were undamaged (Vasconcelos, 1999), we found that 29% of canopy leaves did not present any sign of herbivory. Comparisons of herbivory can also be confounded by stem age and longevity. First, leaf life spans can vary between six months and five years across Amazonian species (Chavana‐Bryant et al., 2019; Reich et al., 1991, 2004) and tend to be longer in the understorey than in the canopy (Reich et al., 2004) and shorter in pioneer species (Galia Selaya et al., 2008) when compared to old‐growth species. Although a 5‐year‐old leaf has more time to accumulate herbivory damage, the scale of this effect is unclear as most herbivory occurs when leaves are developing (Coley, 1983). For instance, if leaves are short lived, then a 6% loss of leaf matter results in a much greater net loss of nutrients to herbivores. Schowalter (2016) report that continual measurement of leaf area loss can provide estimates that are 1–5 times higher than those based on discrete sampling. For instance, using leaf longevity estimates for primary forests from Chavana‐Bryant et al. (2019), and secondary forests from Reich et al. (1991), Reich et al. (2004) suggests a fivefold difference in longevity. If we applied this to our data, the leaf area lost over time could be many times higher in secondary forests than in a primary forest. Such differences in leaf longevity are likely to have an important effect on the impact of herbivory on the carbon and nitrogen cycles. Variation between understorey and canopy herbivory can also be affected by ontogenetic differences in leaf traits (Damián et al., 2018) mature leaves in younger individuals can be more palatable than mature leaves in older individuals of the same species, as the former tend to be less thick and tough than the latter, hence making it easier for herbivore attack (Fortunel et al., 2020). Thus, our static sampling approach may be an underestimate of the actual levels of herbivory in disturbed forests.

### 4.4 Implications and conclusions

Our large‐scale study of canopy herbivory in one of the most biodiverse biomes of the world suggests human disturbance has a low effect on the rates of leaf herbivory. The effect size was small, and the difference between median values of stem‐level chewing severity was just 0.9% between logged and undisturbed forests. These results suggest that the ecological process of leaf herbivory is relatively resilient to human‐driven disturbance despite very high levels of community turnover in the taxa that are key components of this process, including the plants, potential predators such as birds, and the invertebrates (de Castro Solar et al., 2015). However, these snapshot assessments of herbivory incidence also highlight some important areas for new research, as (a) leaf based measures of severity may not reveal the true physiological burden faced by the trees, as they do not include variation in rates of leaf production in different forests, and it remains unclear whether the energetic losses resulting from external‐feeding chewers compare with the impacts of gall‐forming and mining guilds; (b) the top‐down control of herbivory remains poorly assessed, despite global efforts to look at the impacts on external feeders (Howe et al., 2009) and regional assessments (*see review in* Boesing et al., 2017), and (c) it is not clear whether herbivory patterns will be maintained under a changing climate or under higher intensities of human‐driven disturbance.

## CONFLICT OF INTEREST

None declared.

## AUTHOR CONTRIBUTIONS

**Julia Rodrigues Barreto:** Conceptualization (equal); Data curation (lead); Formal analysis (lead); Investigation (lead); Methodology (lead); Visualization (lead); Writing‐original draft (lead); Writing‐review & editing (lead). **Erika Berenguer:** Conceptualization (equal); Data curation (supporting); Formal analysis (supporting); Investigation (supporting); Methodology (equal); Project administration (supporting); Resources (equal); Supervision (supporting); Validation (supporting); Writing‐original draft (supporting); Writing‐review & editing (equal). **Joice Ferreira:** Funding acquisition (equal); Project administration (equal); Writing‐review & editing (supporting). **Carlos Joly:** Funding acquisition; Project administration. **Yadvinder Malhi:** Funding acquisition; Project administration; Writing‐review & editing (supporting). **Marina Maria Moraes de Seixas:** Methodology (supporting); Project administration. **Jos Barlow:** Conceptualization (equal); Formal analysis (supporting); Funding acquisition (lead); Methodology (equal); Project administration (lead); Resources (supporting); Supervision (lead); Validation (equal); Writing‐original draft (supporting); Writing‐review & editing (equal).

### OPEN RESEARCH BADGES

This article has earned an Open Data Badge for making publicly available the digitally‐shareable data necessary to reproduce the reported results. The data is available at http://doi.org/10.6084/m9.figshare.13697443.

## Supporting information

Fig S1

Fig S2

Fig S3

Fig S4

Table S1

Table S2

Supplementary Material

## Data Availability

The original data will be available in an online database by the time the paper is published. The original data that support the findings of this study are openly available in the “figshare” online database at http://doi.org/10.6084/m9.figshare.13697443.
