# Supplementary figures and images for: Assessing invertebrate herbivory in human‐modified tropical forest canopies
Source: Ecol Evol. 2021 Mar 26;11(9):4012–22. doi: 10.1002/ece3.7295 (PMC8093672; doi:10.1002/ece3.7295)

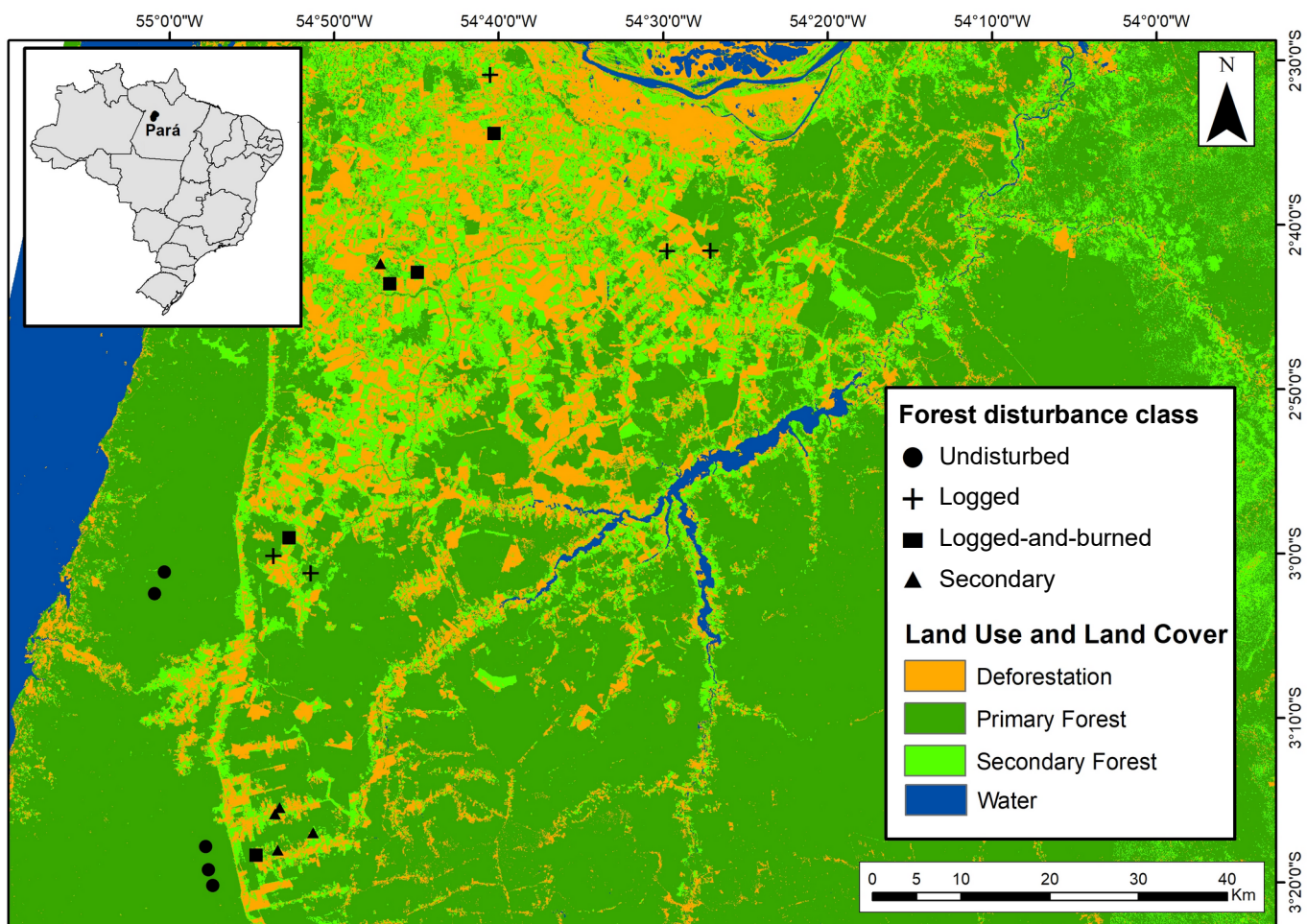

Supplement: Supplementary file 1 — Fig S1 [file ECE3-11-4012-s007.pdf]

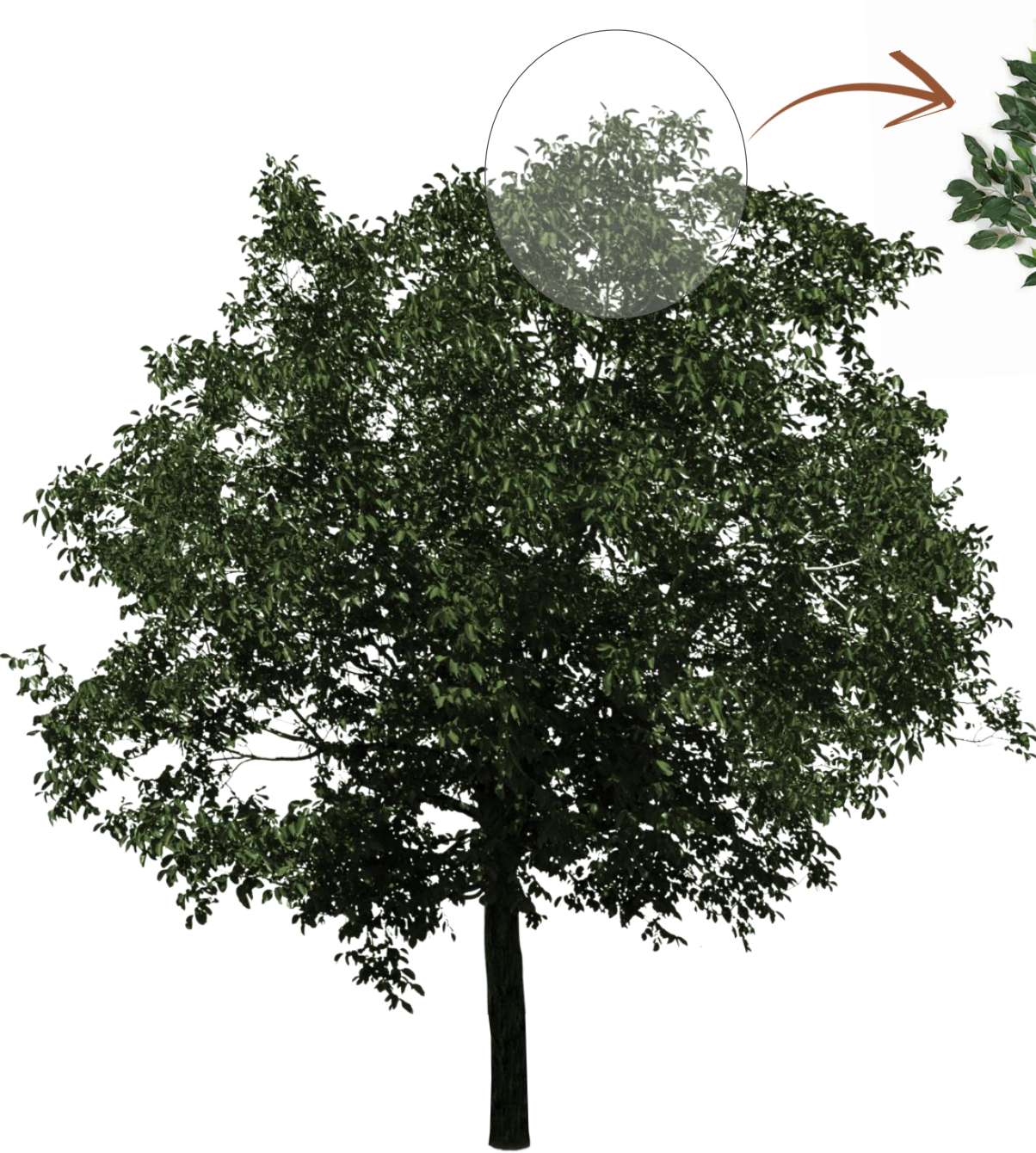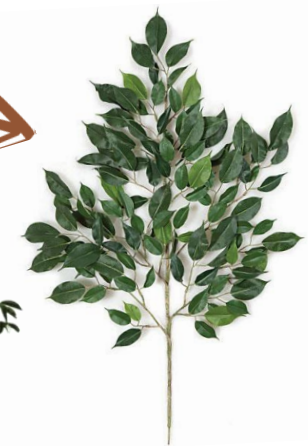

e.g. a branch with  $n = 10$  leaves

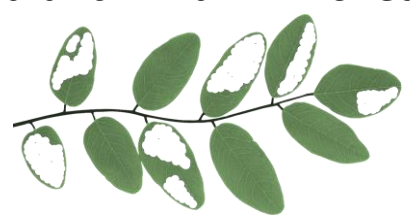

Incidence

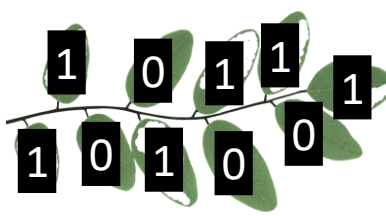

(60% = 6 herbivored leaves/10)

Severity

$$\sum_{i=1}^n H$$

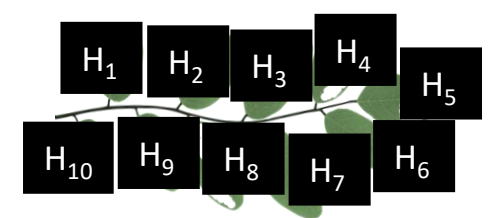

(~35% =  $H_1 + H_2 + \dots + H_{10} / 10$ )

Supplement: Supplementary file 2 — Fig S2 [file ECE3-11-4012-s004.pdf]

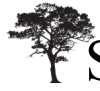

## Stem-level

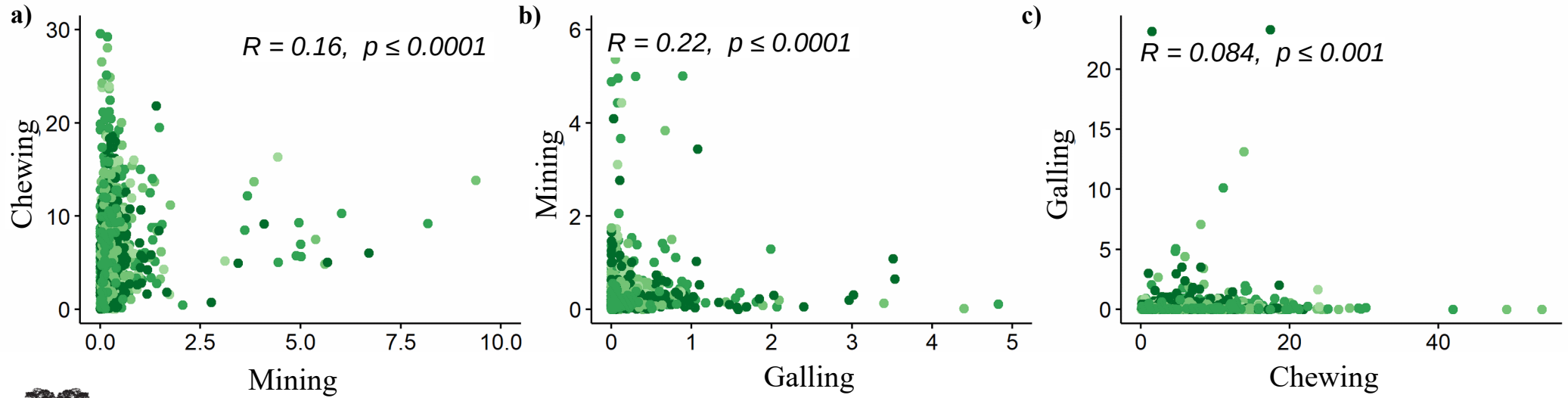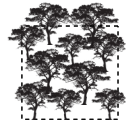

## Plot-level

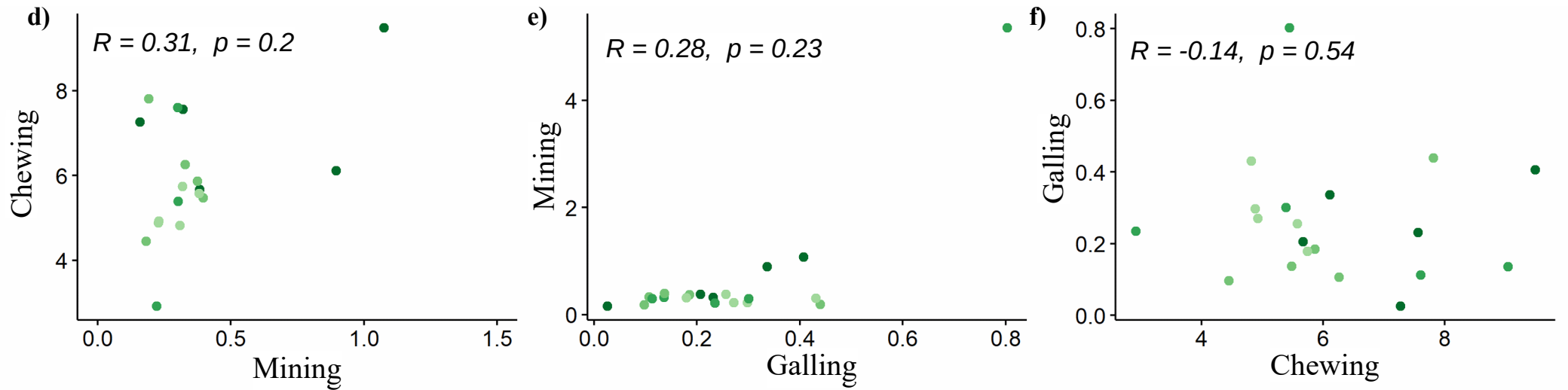

Supplement: Supplementary file 3 — Fig S3 [file ECE3-11-4012-s001.pdf]

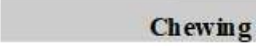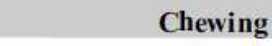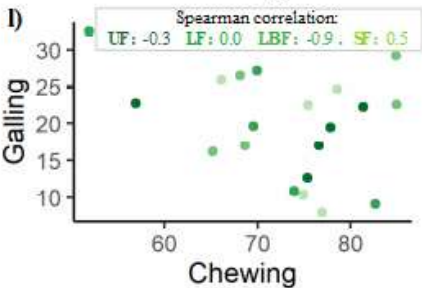

Supplement: Supplementary file 4 — Fig S4 [file ECE3-11-4012-s006.pdf]
