# Supplementary material for: Assessing invertebrate herbivory in human‐modified tropical forest canopies
Source: Ecol Evol. 2021 Mar 26;11(9):4012–22. doi: 10.1002/ece3.7295 (PMC8093672; doi:10.1002/ece3.7295)
Supplement: Supplementary file 5 — Table S1 [file ECE3-11-4012-s003.pdf]

Plot code	Forest class	Latitude	Longitude	Altitude (m.a.s.l.)	Slope (%)	Clay (g/kg)	Distance to edge (m)
261_10	Undisturbed	-3,01872	-55,00517	144	1,41	814,67	297,56
261_9	Undisturbed	-3,04035	-55,01518	139	3,27	824,00	1517,11
363_3	Undisturbed	-3,29672	-54,96369	132	4,56	396,00	2629,36
363_6	Undisturbed	-3,33631	-54,95630	178	4,94	776,00	2344,08
363_7	Undisturbed	-3,32041	-54,96073	163	5,63	721,33	2851,41
112_12	Logged	-2,69311	-54,45194	139	1,76	818,67	316,16
112_8	Logged	-2,69362	-54,49582	143	10,33	818,67	88,06
260_1	Logged	-3,00266	-54,89505	126	5,70	777,33	302,10
260_4	Logged	-3,02022	-54,85698	105	4,46	746,67	304,33
69_8	Logged	-2,51497	-54,67529	135	4,34	662,67	80,75
129_10	Logged-and-burned	-2,72633	-54,77706	113	3,18	794,67	75,27
129_5	Logged-and-burned	-2,71472	-54,74897	97	6,34	582,67	180,90
260_5	Logged-and-burned	-2,98406	-54,87891	107	4,39	720,00	194,32
357_2	Logged-and-burned	-3,30576	-54,91240	158	5,83	754,67	237,34
69_11	Logged-and-burned	-2,57402	-54,67121	101	1,66	478,67	81,53
129_11	Secondary	-2,70589	-54,78637	120	3,73	754,67	75,89
357_3	Secondary	-3,30048	-54,89061	135	6,55	820,00	119,10
357_4	Secondary	-3,28304	-54,85465	71	2,98	88,00	42,70
357_6	Secondary	-3,25727	-54,88871	113	8,33	134,67	82,59
357_9	Secondary	-3,26371	-54,89322	115	14,94	465,33	270,56
