# Supplementary material for: Assessing invertebrate herbivory in human‐modified tropical forest canopies
Source: Ecol Evol. 2021 Mar 26;11(9):4012–22. doi: 10.1002/ece3.7295 (PMC8093672; doi:10.1002/ece3.7295)
Supplement: Supplementary file 6 — Table S2 [file ECE3-11-4012-s002.pdf]

Herbivory form	Response Variable		Spatial autocorrelation	Model			Result (anova)	Observations
	Analysis level	Herbivory level	Moran's *I*	Predictor	Random factors	Seleted error distribution		
Chewing	Stem	Incidence	Non-sig (p= 0.6326)	~ Forest disturbance class (4-level categorical variable)	Site<br>Species<br>Family<br>Stem id (OLRE)	Binomial (glmer)	Non-sig	We modeled the number of chewing-inflicted units in the total number of leaf blades sampled in that stem (chewing incidence) and the proportion of chewing inflicted area in damaged leaves (chewing severity) in that stem as a function of plot forest class. The final model contained site, plant family and species as random factors. Due oversdispersion we also added stem id as an observation level random factor (ORLE).
		Severity	Non-sig (p= 0.7515)		Site<br>Species<br>Family<br>Stem id (OLRE)	Tweedie (glmmTMB)	Signifficative ($p \leq 0.01$)	
	Plot	Incidence	Non-sig (p= 0.2798)		-	Gaussian	Non-sig	Non-nested sampling design as we analyse herbivory at the broader plot level. In a regular linear model, we modeled plot-level weighted chewing incidence and severity (adjusted by plant species dominance per plot) as a function of plot forest class.
		Severity	Non-sig (p= 0.2117)		-	Gaussian	Non-sig	
Mining	Stem	Incidence	Non-sig (p= 0.9139)		Site<br>Species<br>Family<br>Stem id (OLRE)	Binomial (glmer)	Non-sig	As for chewing levels, we modeled mining incidence and severity as a function of plot forest class, with site, plant family and species as random factors. To deal with oversdispersion we also added stem id as an observation level random factor (ORLE).
		Severity	Non-sig (p= 0.4301)		Site<br>Species<br>Family<br>Stem id (OLRE)	Tweedie (glmmTMB)	Non-sig	
	Plot	Incidence	Non-sig (p= 0.6558)		-	Gaussian	Non-sig	Regular linear model containg plot-level weighted mining severity as a function of forest class.
		Severity	Non-sig (p= 0.8803)		-	Gaussian	Non-sig	
Galling	Stem	Incidence	Non-sig (p= 0.9769)		Site<br>Species<br>Family<br>Stem id (OLRE)	Binomial (glmer)	Non-sig	As for chewing and mining, we modeled galling incidence and severity as a function of plot forest class, with site, plant family, species and stem id as random factors.
		Severity	Non-sig (p= 0.5133)		Site<br>Species<br>Family<br>Stem id (OLRE)	Tweedie (glmmTMB)	Non-sig	
	Plot	Incidence	Non-sig (p= 0.07187)		-	Gaussian	Non-sig	Regular linear model containg plot-level weighted galling severity as a function of forest class.
		Severity	Non-sig (p= 0.1116)		-	Gaussian	Non-sig	
