# Supplementary material for: Assessing invertebrate herbivory in human‐modified tropical forest canopies
Source: Ecol Evol. 2021 Mar 26;11(9):4012–22. doi: 10.1002/ece3.7295 (PMC8093672; doi:10.1002/ece3.7295)
Supplement: Supplementary file 7 — Supplementary Material [file ECE3-11-4012-s005.docx]

**S1 Study region**

**Table S1** Forest disturbance classes, geographic coordinates, altitude (m.a.s.l.), slope (%), soil clay content (g/kg) and distance to edge (m) of our 20 study plots.

**Figure S1** Map of the 20 study plots in eastern Amazonia. Plots were distributed in the municipalities of Santarém, Belterra, and Mojuí dos Campos in the Brazilian state of Pará.

**S2 Schematic description of herbivory levels**

**Figure S2** Herbivory sampling design. For each individual stem, a tree climber sampled a branch of mature leaves that were fully exposed to sunlight. We estimated herbivory incidence by assessing the number of leaves affected by each form of herbivory and dividing it by the total number of leaves in the branch. We calculated severity by averaging the percentage of lamina loss across all leaves (including damaged and undamaged ones) in an individual.

**S3 Modelling approach of each response variable**

**Table S2** Modelling table contains model formula, spatial autocorrelation test result, random factors and error distribution for each response variable ordered per analysis

level (stem or plot) and herbivory form with justifications.

**S4 Correlation tests of severity levels between forms of herbivory at stem and plot level**

**Figure S3** Correlation between herbivory incidence at the stem (a-c) and plot level (d-f). Notice that y-axes are not on the same scale.

**S5 Stem and plot level correlation between forms along the gradient of disturbance**

**Figure S4** Stem (panel above) and plot level (panel below) correlations between herbivory forms in each forest disturbance class. Alongside to spearman correlation coefficients, we display p-value significance level with the asterisks as follows: ‘***’ p ≤0.001; ‘**’ p ≤ 0.01; ‘*’ p ≤ 0.05; ‘.’ p ≤ 0.1; and ‘ ’ ns.
